# Overview of a multi-stakeholder dialogue around Shared Services for Health: the Digital Health Opportunity in Bangladesh

**DOI:** 10.1186/s12961-015-0063-2

**Published:** 2015-12-09

**Authors:** Sania Ashraf, Carolyn Moore, Vaibhav Gupta, Anir Chowdhury, Abul K. Azad, Neelu Singh, David Hagan, Alain B. Labrique

**Affiliations:** Bloomberg School of Public Health, Johns Hopkins, Baltimore, USA; mPowering Frontline Health Workers/Jhpiego, Washington, DC USA; Partnership for Maternal, Newborn & Child Health, World Health Organization, Avenue Appia 20, 1211, Geneva, 27 Switzerland; Access to Information (a2i), Dhaka, Bangladesh; Management Information System of the Directorate General of Health Services under the Ministry of Health & Family Welfare, Dhaka, Bangladesh; eSHIFT Partners Network, Geneva, Switzerland

**Keywords:** Collaboration, Consensus, Dialogue, Multi-stakeholder, Policy

## Abstract

**Background:**

National level policymaking and implementation includes multiple stakeholders with varied interests and priorities. Multi-stakeholder dialogues (MSDs) can facilitate consensus building through collective identification of challenges, recognition of shared goals and interests, and creation of solution pathways. This can shape joint planning and implementation for long-term efficiency in health and other sectors. Scaling up the effective use of information and communication technologies (ICTs) requires cohesive strategic planning towards a shared goal. In Bangladesh, the government and partners convened an MSD in March 2015 to increase stakeholder engagement in policymaking and implementation of a national ICT or electronic or mobile health (eHealth or mHealth) strategy, which seeks to incorporate ICTs into the national health system, aligning with the Digital Bangladesh Vision 2021.

**Methods:**

Relevant stakeholders were identified and key priorities and challenges were mapped through key informant interviews. An MSD was conducted with key stakeholders in Dhaka, Bangladesh. The MSD included presentations, group option generation, agreement and prioritization of barriers to scaling up ICTs.

**Results:**

The MSD approach to building consensus on key priorities highlights the value of dialogue and collaboration with relevant stakeholders to encourage country ownership of nationwide efforts such as ICT scale-up. This MSD showed the dynamic context in which stakeholders operate, including those from academia, donors and foundations, healthcare professionals, associations, multilateral organizations, non-governmental organizations, partner countries and the private sector. Through this MSD, participants improved understanding of each other’s contributions and interests, identified existing relationships, and agreed on policy and implementation gaps that needed to be filled. Collaboration among stakeholders in ICT efforts and research can promote a cohesive approach to scaling up, as well as improve policymaking by integrating interests and feedback of different key cross sectoral actors.

**Conclusion:**

MSDs can align stakeholders to identify challenges and solution pathways, and lead to coordinated action and accountability for resources and results. In addition, the MSD template and approach has been useful to guide ICT scale up in Bangladesh and could be replicated in other contexts to facilitate multi-constituency, multi-sector collaboration.

**Electronic supplementary material:**

The online version of this article (doi:10.1186/s12961-015-0063-2) contains supplementary material, which is available to authorized users.

## Background

Multi-stakeholder dialogues (MSD) aim to bring relevant stakeholders with a shared goal to discuss barriers and interests to create mutual understanding and brainstorm a shared course of action towards a productive goal [1, 2]. Research articles have noted the benefit of structured, interactive dialogue that allows stakeholders to better identify challenges and align priorities and action points to assure accountability for resources [3–5]. The utility of MSDs has been demonstrated in policymaking, governance, conflict resolution, and healthcare services as well as in corporate settings [2, 4–6]. Challenges and effective lessons regarding processes and outcomes of MSDs are of interest for those planning to convene similar dialogues with several key stakeholders [2, 4, 6, 7].

Acknowledging the role of multiple partners that work to improve the health of women, children and newborns, the Partnership for Maternal, Newborn and Child Health (PMNCH) sought to promote MSDs as a mechanism through which stakeholder efforts could be made more efficient in translating to measurable outcomes and reliable strategies. To do this, PMNCH has recently developed a key publication – Multi-Stakeholder Dialogues for Women’s and Children’s Health – in collaboration with partners [8]. In addition, the role of digital technologies to aid the achievement of health outcomes has been noted in another publication, Information and Communication Technologies for Women’s and Children’s Health [9]. Guided by the recommendations in these publications, a MSD was designed and conducted in Bangladesh, where information and communication technologies (ICTs) are being used to achieve maternal and child health outcomes.

Technological innovations, increased connectivity, and socio-cultural shifts are leading to increasing use of ICTs in many aspects of development globally, including healthcare delivery and health system efficiency. To achieve an efficient long-term planning process for the use of ICTs for health sector transformation, it is critical to establish national level strategic action by the Ministry of Health and other relevant Ministries through ICT or Telecommunication, as well as policies and frameworks that involve both the private and public sectors [10]. Coordination of stakeholders is crucial to maximize efficient resource use and make progress towards common goals through accountability, an area MSDs can be useful in facilitating [1]. The WHO National eHealth Strategy Toolkit emphasizes the need for national level planning, stakeholder engagement, and ongoing monitoring and evaluation to propagate national strategy implementation processes for the use of ICTs to evaluate project maturity for readiness to scale up mHealth [1, 2].

In Bangladesh, the rapid rise of household mobile phone ownership (up to 89% in 2014) has led to 125 million mobile phone subscribers and 46 million internet users [11]. High access and improved internet connectivity provide a clear opportunity for ICTs to improve healthcare access, use and surveillance in Bangladesh [12]. This has been demonstrated by carefully evaluated research studies and a proliferation of mHealth and eHealth initiatives led by numerous stakeholders, the majority of which have been used to improve maternal and child healthcare outcomes [13–15]. eHealth initiatives have proliferated through private organizations, mobile phone companies and non-government organizations leading to services like e-Clinic, D.Net (Development Research Network), Telemedicine Reference Center Ltd., BRAC Manoshi, and Aponjon, among many others [16, 17]. Making major gains in the national health system by leveraging the power of ICTs is a stated goal as exemplified in the ICT Policy 2009 and Strategic Priorities of Digital Bangladesh Report 2011 [12].

The Government of Bangladesh is currently adopting an ambitious national ICT/eHealth strategy to incorporate ICTs into their health system, aligning with the Digital Bangladesh Vision 2021 [12]. The Directorate General of Health Services (DGHS) has focused on launching innovative networked ICT applications to improve quality of governance of delivery systems, accountability and archiving of health data to progress towards a digitized health system at the national to grassroots level [16]. As part of the Digital Bangladesh Vision 2021, initiatives to develop eHealth standards and shared health records are underway which will facilitate the provision of Universal Health Coverage, an important aspect of the post-2015 goals [18, 19]. Details and progress of the health information system have been recently published elsewhere [18]. From the Prime Minister’s Office of Bangladesh, the UNDP and USAID-supported organization Access to Information Programme (a2i) is facilitating adoption of ICTs within the government for service delivery improvement, decision making and policy formulation [20]. It leads efforts to create new eServices, strengthen existing ones, and ensure interoperability across various platforms through a whole-of-government approach [18].

Previous evaluations have highlighted important challenges in the scaling up of ICTs in Bangladesh [16]. To address many of the known and emerging barriers and understand the collective priorities of key stakeholders during the scaling up of ICTs for healthcare by the Government, we identified Bangladesh as a unique setting to conduct a MSD.

This paper captures the major findings and reflects on the success of a day-long MSD entitled “Shared Services in Health – The Digital Opportunity” that was held on March 18, 2015, in the Prime Minister’s Office in Dhaka, Bangladesh. The MSD was convened by the DGHS of the Ministry of Health & Family Welfare, and the a2i Programme of the Prime Minister’s Office, supported and funded by the PMNCH. The Global mHealth Alliance at Johns Hopkins University also supported this MSD, while mPowering Frontline Health Workers provided documentation support.

## Methods

### Establishment of a planning group

A planning group comprising of PMNCH, the Global mHealth Initiative at the Johns Hopkins School of Public Health, a2i, and the Management Information System department of the DGHS was assembled to identify key stakeholders and initial goals for the MSD. Partners from Gesellschaft für Internationale Zusammenarbeit also provided technical expertise. These partners are leading the national ICT scale up in Bangladesh and it was crucial to engage these parties at the planning stage. This group outlined the objectives of the MSD, and ensured that the design was effective, timely and appropriate, and that it aligned with progress made to roll out the eHealth strategy and align digital platforms for shared services.

### Initial assessments

Preparation and situational assessment are critical elements of the MSD process as described in the PMNCH guide [8]. Prior literature and existing knowledge of the ongoing initiatives was used to inform a series of one-to-one stakeholder interviews to assess their roles and priorities [13, 15, 21–23]. These interviews were flexible and engaged the stakeholder to describe their role in the ICT scene in Bangladesh as well as their roles in and perceptions of the nationwide scale up plans. They were asked if they would be interested in participating on an MSD and what specific agenda they would like to see highlighted during the meeting.

Based on the needs and the emerging objective of the MSD, the planning group collectively decided that the convener of the meeting should be a2i and the DGHS given their leadership and executive role in the ICT scale up.

### Facilitator training

A PMNCH-funded MSD facilitator’s training was held in Maputo, Mozambique, from February 14–19, 2015, to train facilitators to plan, conduct and follow-up on large MSDs [24]. Members of the Bangladesh MSD team attended and received training on skills such as conflict resolution, negotiation and facilitation, and preparation, including stakeholder and relationship mapping and analysis.

### MSD goals

The conveners identified several areas of discussion, including the facts that (1) many groups of ICT users and implementers in Bangladesh are increasingly capturing digital data, some of which may be useful to strengthen national health information systems; (2) technical channels for the efficient exchange of information do not exist; and (3) appropriate incentives, quality control standards, and accountability measures have not been defined.

Focusing on reaching agreement on key risk factors that would hinder scaling ICTs in Bangladesh, the MSD aimed to enable consensus on key factors having an impact on the national eHealth vision and to develop options for addressing implementation challenges. The MSD was guided by key dimensions of focus while scaling up ICT as detailed in the PMNCH ICT guide [9]. Focusing on reaching agreement on key risk factors for using ICTs in Bangladesh, the MSD will (1) identify broad health system domains where ICTs can contribute; (2) identify barriers and challenges to digital collaboration across stakeholders, including the government, research organizations and non-government organizations; (3) identify mechanisms to address these challenges and strategically align existing and future ICT efforts to maximize efficient information sharing, linkages and use towards improved health outcomes; and (4) maximize efficient information sharing, data quality, access and use during national scale up of health informatics strategies.

### MSD format and facilitation

A total of 43 participants attended, representing seven constituencies including academic, research and teaching institutions, donors and foundations, healthcare professionals associations, multilateral organizations, non-governmental organizations, partner countries, and the private sector working in the eHealth and mHealth arena in Bangladesh (Table 1). Leadership of the Government of Bangladesh was represented by the attendance of distinguished members such as the Principal Secretary, the Secretary of the Ministry of Health and Family Welfare, the former Secretary of the Health and Family Welfare, and the Executive Director of the Bangladesh Computer Council and Director General (Administration) of Prime Minister’s Office. a2i provided neutral facilitation of the participatory process using the well-structured flexible format detailed below.

**Table 1:** **Stakeholder domains represented**

**Domains**	**Participants, n (%)**
Academic	6 (12)
Donors and foundations	5 (10)
Healthcare sector	2 (4)
Multilateral organisations	11 (22)
Research and teaching institutions	4 (8)
Government of Bangladesh	11 (22)
Non-governmental organizations	4 (8)
Private sector	7 (14)

The MSD opened with short presentations by a2i highlighting the National eHealth vision and the progress, penetration and opportunity of ICTs in Bangladesh. This was followed by nearly 5 hours of facilitated dialogue that allowed the group of stakeholders to prioritize key areas of concern and generate options and recommendations in each of these areas. This dialogue process was based on a Mutual Gains Approach that emphasizes negotiation and consensus building for shared benefit of the group [8]. The key principles of this approach are to (1) prepare effectively by focusing on stakeholders’ interests and best alternatives to a negotiated agreement and by generating initial proposals for mutual gains; (2) in value creation, begin by exploring needs and interests, not by stating positions; (3) to find potential mutual gains, use no-commitment brainstorming to develop options and proposals that might meet both one’s own needs and interests and those of other stakeholders; (4) seek maximum joint gains before moving to value distribution (i.e. making commitments and compromise on deciding who gets what); (5) when distributing value, find mutually acceptable criteria for dividing joint gains; (6) in follow-through, ensure that agreements will be sustainable by committing to continuing; and (7) communication, joint monitoring, contingency planning and dispute resolution mechanisms.

Participants mapped the numerous and diverse eHealth initiatives active in Bangladesh through a small group brainstorming exercise. Then, a pile sort exercise was used to identify challenges that had been overcome to date in the implementation and scale-up of eHealth in Bangladesh, as well as current and future challenges yet to be overcome. In small groups, participants wrote responses on cards, which were then posted on a wall. The large group sorted the cards into common groups to determine commonly identified challenges (Additional file 1). This hands-on exercise was noted to be an effective way to engage participants who had not participated as actively in the large group discussions.

In small groups, participants discussed specific areas identified in the pile sort exercise. Participants were not grouped by facilitators, but instead chose the area of greatest interest to them. Each group identified challenges to ICT scale-up and recommended next steps to overcome these challenges. The day ended with a summary of the discussion and agreement on the way forward.

## Results

In-depth interviews prior to the MSD were used to map the landscape of stakeholders working in eHealth and mHealth in Bangladesh. This mapping helped the planning team understand the nature of the key stakeholders, their priorities and how that would shape the goal of the MSD. It was critical to introduce the concept of a MSD one-on-one and engage multiple stakeholders in a discussion to identify known challenges and opportunities that could be addressed in the MSD design.

The multi-stakeholder dialogue successfully brought together important stakeholders from multiple domains. The facilitator used techniques from the Mutual Gains Approach to emphasize negotiation and consensus building based on common interests rather than their positions. Activities such as group mapping and pile sorting enabled the groups to emphasize their interests and concerns without identifying their source in the larger discussion. Participants from multiple domains were purposively assigned to groups to enable sharing of concerns and priorities across organizations and collectively brain storm solutions. Some of the key findings from the meeting are presented.

### Current initiatives

Multi-stakeholder groups reported that current eHealth and mHealth initiatives covered patient care, provider education and training, behaviour change interventions, and facility management applications, among others. The participants identified policies and initiatives, collective accomplishments, infrastructure developments, health systems changes, and programmatic initiatives operating locally, as well as global changes that affect Bangladesh. This demonstrated the large number of technical areas and implementation activities where ICT and eHealth are in use.

### Challenges

The key challenges identified by this multi-stakeholder group for scaling up ICT in Bangladesh included policy and legal framework, interoperability, IT infrastructure, and capacity, monitoring and evaluation. Participants also acknowledged that issues of quality and equity remained challenges, and should be considered within each solution area. Each group presented multifaceted solution routes from its discussion, including new policies, adoption of current policies, stakeholder engagement and collaboration, training, and programmatic and implementation changes. Priority areas and recommendations are discussed. Priority areas and recommendations for ICT scale-up for health services in Bangladesh are included in Additional file 1.

Following the group presentations, the large group engaged in a discussion of contributing factors necessary to ensure successful scale-up of ICTs and support the recommendations suggested by the group.

### Recommendations

These were broadly grouped into institutional and stakeholder leadership, policy, quality and coverage of care, and collaboration and stakeholder engagement.

#### Institutional and stakeholder leadership

Participants called for an administrative leadership body within the government with the mandate to oversee ICT across all sectors. They recognized the leadership that the Ministry of Health and Family Welfare has shown on this issue but also pointed out that they were not mandated to do so under any formal parliamentary regulations. Participants felt that existing departments would not be able to keep up with the pace of capacity building needed for ICT oversight, and identified a need for institutions with the capacity to oversee eHealth and mHealth in a comprehensive way. Political and bureaucratic leadership is required to allow integration of eHealth into the health system and promote a professional culture that embraces the use of eHealth and its data, and capacity building is needed to ensure that health sector policymakers and implementers comprehend and utilize electronic health information systems. They acknowledged the need for responsibility to be taken beyond government, and identified several action areas that implementers could begin to address immediately.

#### Policy

Recommendations for national and global policy emerged from the MSD. Participants recommended that policy and government oversight be used to ensure accountability, transparency, neutrality, and environmental responsibility in the scale-up of ICTs. They also identified a need for a regulatory framework to standardize and guide architecture, data, and cohesive implementation. Since health in post-2015 development goals will necessitate the tracking of all citizens, Civil Registration and Vital Statistics should be an important focus area as its impact will go into areas beyond the health sector, including social security, education and civil service sectors [21, 23]. Participants suggested that the integration of health information systems and eHealth in health systems should be a key component of the new Global Strategy for Women’s, Children’s and Adolescents’ Health for achievement of post-2015 Sustainable Development Goals particularly to support women’s, children’s and adolescents’ health.

#### Quality and coverage

To improve quality and coverage of care in line with the Universal Health Coverage vision, participants recommended that eHealth be used to create an environment where patients and providers are satisfied with the type and quality of healthcare, using an interoperable electronic health information system [22]. Issues of equity and quality should be examined in the implementation and evaluation of all eHealth initiatives with an overall goal to achieve universal coverage of high-quality and affordable care, thus reducing the time, cost, and number of visits required of a patient to access high-quality, timely care.

#### Stakeholder engagement

Shared services will require meaningful engagement of all stakeholders. This includes communities and citizens, and participants expressed the need for clear citizens’ responsibilities in regulatory framework (for example, requiring citizens to maintain and update elements of their electronic health records). Participants praised the MSD structure and called for continued dialogue and coordinated action to arrive at solutions.

#### Key initiatives

Participants recommended several initiatives to take forward their recommendations, including improved used and quality assurance of telemedicine, shared health records, health insurance coverage, initiatives to improve and expand infrastructure, unique IDs for citizens, projects to improve service delivery and link registration to service delivery, education, training, and career pathways to improve ICT capacity in program, health sector, and Ministry staff, strengthening routine health information systems, and conferences to strengthen collaboration and dissemination of information related to eHealth and mHealth. The group agreed on five overall objectives to summarize their recommendations: (1) improve universal coverage and quality of preventive, primary, and clinical care; (2) improve cross-sector coordination for better coordination of care; (3) build capacity across multiple levels of providers; (4) empower citizens to improve knowledge and service utilization; and (5) harmonize and standardize multiple initiatives.

To close the meeting, the Secretary of the Ministry of Health and Family Planning emphasized the role of eHealth to strengthen the national health system. The endorsement of joint work by the Principal Secretary of the Prime Minister’s Office and the Secretary of the Ministry of Health and Family Welfare encouraged a2i and the Ministry to sign a Memorandum of Understanding to launch joint initiatives on eHealth and mHealth and involve private sector partners. There is a need for improved management and leadership for sustainable engagement to use resources efficiently. While eHealth technologies cannot fulfil all needs, scaling up of effective innovations has the potential to improve health services by mitigating poor human resources in hard-to-reach areas.

## Discussion

In the context of ICT scale-up in Bangladesh, despite the cooperation and consensus demonstrated through the MSD, there remain complex barriers for stakeholders when acting on emerging recommendations. This MSD was effective in articulating stakeholders’ priorities and determining critical areas for action, but strong leadership will be needed to implement the changes recommended by the participants and to guide the use of ICTs in the long term. The convener (a2i) will coordinate action to ensure that recommendations are taken forward, but resource and capacity constraints were recognized as a concern. Participants displayed a high level of commitment to the recommendations of the MSD, but noted the lack of an institution with the specific mandate to coordinate future stakeholder engagement. Stakeholders called for additional opportunities to collaborate through a national meeting, working groups, or other venues. They recognized that government, organizational, and community collaboration would be necessary to implement the recommendations from this MSD.

The findings are consistent with other assessments conducted to evaluate the challenges facing eHealth projects in Bangladesh [16, 17]. It highlights the need for organized cooperation among diverse participating parties to contribute towards training of users and providers to raise acceptance. Technical aspects, such as the need for an interoperable system, adequate trouble shooting and effective measures to implement the national policy, emerged as findings from this MSD. Future interventions to increase the successful scaling up of ICTs can involve the participating organizations to rely on cohesive action plans. Improving the quality of user-centric technology is important to increase the acceptability of eHealth technologies and further research is needed to collate aspects that have been tested by various organizations [24]. Effective use of ICTs can educate, empower and connect vulnerable populations to health services in low-income settings for a variety of health services ranging from maternal, neonatal health, immunization, and chronic health, as demonstrated through recent studies [25–27].

MSDs have been used to achieve legitimacy and accountability through multilateral agreements and more effective leadership and monitoring mechanisms in developing and developed countries [6, 30]. It has also been used in corporate organizations to emphasize efficiency through issue-focused problem solving [4, 28, 29] . Several elements were instrumental in the use of the MSD format in Bangladesh and are recommended for future MSDs in other areas. These include (1) engaging stakeholders to prepare them for the meeting and share their perspectives with the facilitators prior to the MSD; (2) ensuring that participants represented key domains relevant in the context of the topic and were knowledgeable about day-to-day implementation of ICT programs; (3) neutral facilitation that allows emergence of shared action plans and promotes ownership through active participation and representation of attendees, including the use of hands-on activities and group discussions to engage all participants; and (4) sharing convening by multiple organizations, with care taken to create a neutral space for open discussion, without a perceived organizational agenda.

Future MSDs should engage a similar variety of experienced stakeholders, with the potential to increase participation from key participants in leadership roles from relevant constituencies. This MSD had some limitations. Although several stakeholder interviews were conducted prior to the meeting, we could not reach all the constituencies represented in the MSD prior to the meeting. More interaction with some of the attending participants would have allowed the planning group to identify their priorities and include them in the agenda to maximize their engagement during the meeting. While the 1-day MSD was successful in identifying key issues and developing dialogue, a longer meeting (e.g. 1.5 or 2 days) would have allowed time for more in-depth discussions, introductions of all participants, and an in-depth discussion on engagement plans for follow-up and accountability. MSDs in other sectors have been planned in Bangladesh, including an MSD on Financial Inclusion specifically for social safety nets in June 2015, and future MSDs in education, agriculture, land, law and order, and other sectors. Follow-up on the use of MSDs in Bangladesh can be documented to learn about successes and challenges in different working groups.

While the MSD model was effective in generating consensus and identifying priority areas for action, specific solutions will need to be developed and adopted as longer term follow-up to the MSD. This will require continued stakeholder engagement and ongoing coordination on the part of the conveners. Long-term engagement with stakeholders can be complex based on the multifaceted nature of the recommendations presented and the variety of stakeholders involved. Working groups can help to build ownership of specific efforts, but selection of these groups may require further engagement efforts through follow-up dialogues and dissemination efforts. Further consensus on action items associated with each recommendation is required, as is allocation of funds and/or resources to achieve the goals identified in the MSD.

As part of its Strategic Plan for 2016–2020, the PMNCH is committed to enhancing capacity for multi-stakeholder participation, alignment and action at the country, regional and global levels, including the identification, synthesis and dissemination of replicable best practices to facilitate the implementation of effective multi-stakeholder partnerships in countries. WHO and PMNCH recently successfully convened another MSD on sexual, reproductive, maternal, newborn, child and adolescent health in the capital Lusaka [30]. PMNCH has convened a group of experts to contribute to a knowledge summary on multi-stakeholder dialogues. The purpose of this knowledge summary is to introduce MSD best practice and illustrate how MSDs can be utilized in support of existing multi-stakeholder efforts and country platforms for the implementation of the Sustainable Development Goals and the updated Global Strategy for Women’s, Children’s and Adolescents’ Health (2016–2030).

## Conclusions

Collaborative dialogues with multiple stakeholders to create ownership of implementation agendas are recognized as effective strategies in both low-income and developed country settings [2, 7]. In Bangladesh, MSD participants identified infrastructure, policy and legal framework, interoperability, and capacity, monitoring and evaluation as challenges to the scale-up of ICTs for shared services in health, and proposed varied solutions in each area. There is high potential to replicate the MSD model to reach consensus for planning complex large scale implementation across various sectors such as ICT, education, agriculture, and civil services. This MSD facilitated collaboration among various public and private stakeholders who had not previously collaborated formally, and prompted the planning of MSDs in other sectors. Such MSDs should be seen as a part of an ongoing set of activities to ensure cohesive action towards a coordinated goal. The MSD model could be used to facilitate the use of ICTs in many countries where there is an active or emerging eHealth landscape. While many of the key issues identified by participants are prevalent in eHealth and ICT implementation worldwide, the specific considerations and recommendations are expected to vary based by sector and geographic area. When replicating the MSD model, it is important to include success factors described above, particularly the preparatory and follow-up work described in the PMNCH guide [8]. The mix of methods in this guide encourages participation from participants with varied working styles, including any participants who are hesitant to speak openly in large group discussions. The MSD template could be replicated in other contexts to facilitate multi-constituency, multi-sector collaboration for long-term engagement and to develop effective implementation agendas with shared ownership.

## Additional file

Additional file 1:
**Priority areas and recommendations for ICT scale up for health services in Bangladesh, 2015.** (DOCX 38 kb)

## Abbreviations

a2i: Access to information
MSD: Multi-stake holder dialogue
DGHS: Directorate General of Health Services
ICT: Information and communication technology
PMNCH: Partnership for Maternal, Newborn and Child Health
