# Supplementary material for: Overview of a multi-stakeholder dialogue around Shared Services for Health: the Digital Health Opportunity in Bangladesh
Source: Health Res Policy Syst. 2015 Dec 9;13:74. doi: 10.1186/s12961-015-0063-2 (PMC4673719; doi:10.1186/s12961-015-0063-2)
Supplement: Additional file 1: — Priority areas and recommendations for ICT scale up for health services in Bangladesh, 2015. (DOCX 38 kb) [file 12961_2015_63_MOESM1_ESM.docx]

Additional file 1: Priority areas and recommendations for ICT scale up for health services in Bangladesh, 2015

| Priority Area | Infrastructure | Policy and Legal Framework | Interoperability | Capacity, Monitoring, and Evaluation |
| --- | --- | --- | --- | --- |
| Definition of success | A person in need of a health service has affordable and accessible service. | A national eHealth policy with the goals of strengthening health systems, achieving universal health coverage, and achieving Sustainable Development Goals (SDGs). | Coordination of varied eHealth initiatives active in Bangladesh to allow systems, data, and processes to be compatible and to build upon each other. | Improved training for clinical and public health professionals to effectively integrate eHealth into their work; institutional ability to plan, implement, and oversee eHealth programs; with improved monitoring and evaluation |
| Immediate Recommendations | 1. Improve physical infrastructure and develop ‘fall back’ or second operator options to reach a given area.  2. Distribute bandwidth based on need, not only population or market potential.  3. Improve bandwidth reliability so that coverage is accessible, available, and affordable, at all times, not only during facility hours and regardless of usage level.  4. Decentralize maintenance for data systems; include fall-back options to provide continued service during maintenance.  5. Improve inter-agency or inter-department cooperation to improve infrastructure and reduce policy gaps. | 1. Create a robust eHealth policy that addresses: data privacy, confidentiality, and security; telemedicine; intellectual property; stakeholder participation; electronic health records; and citizens’ responsibilities.  2. Develop an eHealth funding mechanism.  3. Create a regulatory body or official National eHealth Council to oversee stakeholders and enforce issues around patient rights; accountability and transparency; and quality. | 1. Complete existing work to assign each citizen a unique ID, and create IDs for facilities and providers.  2. Put into practice the citizen core data structure, a standardized way to collect citizen data.  3. Implement and adopt the existing standard coding system.  4. Improve capacity for interoperability, including maintenance.  5. Develop institutional frameworks and processes to ensure and oversee interoperability  6. Ensure API compliance and compatibility in all systems. | 1. Develop an ICT Competency Checklist to define skills needed for staff.  2. Develop community of practice around eHealth and an open eHealth project inventory.  3. Require M&E plans for all new eHealth initiatives. |
| Future Recommendations | *All recommendations presented as immediate* | *All recommendations presented as immediate* | 1. Require health providers to contribute to a shared health record that also ensures patient privacy  2. Define responsibility for birth registration and ID creation, so that institutional roles and expectations are clear.  3. Implement legal reform for marriage, divorce, and adoption, to capture currently missing ID data. | 1.Develop training curriculum for ICT competency to introduce into existing training curriculum;  Train all staff to develop institutional capacity and promote a ‘data culture’, including training on a local level.  2. Perform data audits to increase data reliability and quality.  4. Strengthen data at district level.  5. Create a forum for regular stakeholder dialogue and collaboration; convene a national eHealth curriculum task force.  6. Create new posts to fill gaps in institutional capacity around ICT and eHealth.  7. Promote local community engagement in eHealth initiatives.  8. Increase funding for research and evaluation of eHealth interventions. |
